# Dry Operations

**Published:** 1890-04

**Authors:** 


DRY OPERATIONS have been lately advocated by Landerer, of Leipsic, among others. His reasons for rendering operations as dry as possible are not without weight. He points out that the employment of all sorts of antiseptics may be dangerous under certain conditions, and that it is advisable to avoid them as much as possible. The employment of sterilized fluids in large quantities is inconvenient and messy, and there are many advantages in keeping the field of operation quite dry. The essential part in the dry method is, immediately after the first incision, to keep every part of the operation field, not for the moment under the knife, under pressure by means of antiseptic gauze—dry sublimate gauze is what he recommends. By this means the wound can be closed more quickly, as the plugging will have arrested the bleeding, and drainage will frequently be avoided altogether. The suturing then takes place, after which a moderately firm compress is applied. In this way the patient is less messed and cooled, there is less loss of blood, and above all, no germs can enter from the moment the plugging is applied. The bleeding is more quickly arrested, and the duration of the operation is shortened, while at the same time healing is rendered more rapid and certain.—*The Medical Press.*

